# Perceived costs as drivers of wildlife management preferences in rural Tanzanian communities

**DOI:** 10.1111/cobi.70251

**Published:** 2026-03-09

**Authors:** Christian Kiffner, Justin Raycraft, Reilly Becchina, Danielle Bettermann, Stephen Koester, Elana Kriegel, Kiana Lindsay, Edwin Maingo Ole, Emily Ramirez, Bryan Spizuco, Neil H. Carter

**Affiliations:** ^1^ Leibniz Centre for Agricultural Landscape Research (ZALF) Müncheberg Germany; ^2^ Center for Wildlife Management Studies School for Field Studies Karatu Tanzania; ^3^ Department of Human Behavior, Ecology and Culture Max Planck Institute for Evolutionary Anthropology Leipzig Germany; ^4^ Faculty of Life Sciences, Thaer‐Institute of Agricultural and Horticultural Sciences Humboldt‐Universität zu Berlin Berlin Germany; ^5^ Department of Anthropology University of Lethbridge Lethbridge Alberta Canada; ^6^ Department of Anthropology University of Maryland College Park Maryland USA; ^7^ Department of Environmental Initiative Lehigh University Bethlehem Pennsylvania USA; ^8^ Department of History and Philosophy University of Münster Münster Germany; ^9^ Muhlenberg College Allentown Pennsylvania USA; ^10^ Department of Animal Science Cornell University Ithaca New York USA; ^11^ Icahn School of Medicine at Mount Sinai Mount Sinai Hospital New York New York USA; ^12^ University of San Diego San Diego California USA; ^13^ School for Environment and Sustainability University of Michigan Ann Arbor Michigan USA; ^14^ Department of Agricultural Extension and Community Development Sokoine University of Agriculture Morogoro Tanzania; ^15^ Lafayette College Easton Pennsylvania USA; ^16^ The Rochester Institute of Technology Rochester New York USA

**Keywords:** human–wildlife coexistence, human–wildlife conflict, human–wildlife interactions, large carnivores, large herbivores, tolerance, carnívoros grandes, coexistencia humano‐fauna, conflicto humano‐fauna, interacciones humano‐fauna, herbívoros grandes, tolerancia, 人兽互动, 人兽共存, 人兽冲突, 大型食肉动物, 大型食草动物, 容忍度

## Abstract

Effectively managing human–wildlife interactions is crucial for fostering coexistence on shared landscapes. Management options are most effective when aligned with the preferences of people directly affected by wildlife, yet little is known about how socioecological factors influence these preferences. Integrating responses from 680 rural residents of northern Tanzania and remotely sensed data, we parameterized a Bayesian hierarchical model to test predictions of the hazard‐acceptance model. We estimated how perceived costs and benefits, distance to protected areas, and the human footprint index mediate preferences for managing (preventing damage, compensating damage, reducing populations, and doing nothing) interactions with herbivore (elephant, giraffe, buffalo, zebra, wildebeest, and impala) and carnivore (lion, hyena, leopard, cheetah, honey badger, and jackal) species. Most respondents preferred management options that supported coexistence: prevention (41.9%), no management (38.0%), and compensation (11.1%). In contrast, population reduction (9.0%) was least preferred but more frequently selected for carnivores (13.4%) than herbivores (5.3%). Perceived costs strongly influenced management preferences. Respondents perceiving tangible costs were more likely to prefer prevention (posterior mean: 0.57 [95% credible interval 0.00 to 0.99]) over compensation (0.07 [0.00 to 0.66]) or population reduction (0.16 [0.00 to 0.87]), whereas those not perceiving costs leaned toward no management (0.40 [−0.74 to 1.78]). Though perceived benefits were less influential than costs, respondents associating species with intangible (0.10 [0.00 to 0.74]) or tourism benefits (0.06 [0.00 to 0.63]) were less likely to support population reduction than those perceiving no benefits (0.12 [0.00 to 0.82]). Distance to protected areas and the human footprint index had weaker, inconsistent effects, but random intercepts indicated substantial village–village variation in preferred management options. Our results suggest that conservation strategies should primarily address wildlife‐related costs and foster coexistence by more equitably distributing benefits. A possible strategy could include investing tourism revenues into comanaged, locally tailored damage prevention measures.

## Abstract (es)

Costos percibidos como factores de preferencia en el manejo de fauna en las comunidades rurales de Tanzania

**Resumen:**

La gestión eficaz de las interacciones entre humanos y fauna es fundamental para fomentar la coexistencia en paisajes compartidos. Las opciones de gestión son más eficaces cuando se ajustan a las preferencias de las personas directamente afectadas por la fauna silvestre, pero se sabe poco sobre cómo influyen los factores socioecológicos en estas preferencias. Integramos las respuestas de 680 residentes rurales del norte de Tanzania y datos obtenidos por teledetección y parametrizamos un modelo jerárquico bayesiano para comprobar las predicciones del modelo de aceptación del riesgo. Estimamos cómo los costos y beneficios percibidos, la distancia a las áreas protegidas y el índice de huella humana median las preferencias para gestionar (prevenir daños, compensar daños, reducir poblaciones y no hacer nada) las interacciones con especies herbívoras (elefantes, jirafas, búfalos, cebras, ñus e impalas) y carnívoras (leones, hienas, leopardos, guepardos, tejones mieleros y chacales). La mayoría de los encuestados prefirió opciones de gestión que apoyaban la coexistencia: prevención (41.9%), no gestión (38.0%) y compensación (11.1%). Por el contrario, la reducción de la población (9.0%) fue la menos preferida, pero se seleccionó con más frecuencia para los carnívoros (13.4%) que para los herbívoros (5.3%). Los costos percibidos influyeron considerablemente en las preferencias de gestión. Los encuestados que percibían costos tangibles se mostraban más propensos a preferir la prevención (media posterior: 0.57 [intervalo de credibilidad del 95%: 0.00 a 0.99]) frente a la compensación (0.07 [0.00 a 0.66]) o la reducción de la población (0.16 [0.00 a 0.87]), mientras que los que no percibían los costos se inclinaban por no gestionar (0.40 [‐0.74 a 1.78]). Aunque los beneficios percibidos influían menos que los costos, los encuestados que asociaban las especies con beneficios intangibles (0.10 [0.00 a 0.74]) o turísticos (0.06 [0.00 a 0.63]) eran menos propensos a apoyar la reducción de la población que aquellos que no percibían ningún beneficio (0.12 [0.00 a 0.82]). La distancia a las áreas protegidas y el índice de huella humana tuvieron efectos más débiles e inconsistentes, pero las intersecciones aleatorias indicaron una variación sustancial entre pueblos en cuanto a las opciones de gestión preferidas. Nuestros resultados sugieren que las estrategias de conservación deben abordar principalmente los costos relacionados con la fauna silvestre y fomentar la coexistencia mediante una distribución más equitativa de los beneficios. Una posible estrategia podría incluir la inversión de los ingresos del turismo en medidas de prevención de daños cogestionadas y adaptadas a las necesidades locales.

## 坦桑尼亚农村社区中感知成本对野生动物管理偏好的驱动作用

**【摘要】** 有效管理人兽互动关系对促进共享景观中的人兽共存至关重要。当管理方案符合受到野生动物直接影响人群的偏好时, 其管理效果最为显著, 但目前尚不清楚社会生态因素如何影响人们的这些偏好。本研究整合对坦桑尼亚北部680名农村居民的访问调查及遥感数据, 构建了参数化的贝叶斯层次模型, 以检验危害‐接受模型的假设。我们评估了人们感知的成本及收益、到保护区的距离, 以及人类足迹指数如何介导与食草动物(包括大象、长颈鹿、水牛、斑马、角马和黑斑羚)及与食肉动物(包括狮子、鬣狗、豹、猎豹、蜜獾和豺)互作的互动管理策略偏好, 如预防危害、补偿危害、削减种群, 以及不干预。大多数受访者倾向支持共存的管理方案, 即预防(41.9%)、不干预(38.0%)、补偿(11.1%)。相比之下, 种群削减方案虽最不受欢迎(9.0%), 但针对食肉动物的选择率(13.4%)显著高于食草动物(5.3%)。人们的感知成本显著影响其管理偏好。感知到具体成本的受访者更倾向于选择预防措施(后验均值:0.57 [95%置信区间0.00至0.99]), 而非补偿措施(0.07 [0.00至0.66])或种群削减措施(0.16 [0.00至0.87]), 而未感知到成本的受访者则倾向于不干预(0.40 [‐0.74至1.78])。尽管感知收益的影响低于感知成本, 但相比于未感知到收益的受访者(0.12 [0.00至0.82]), 认为物种可带来无形收益(0.10 [0.00至0.74])或旅游收益(0.06 [0.00至0.63])的受访者对种群削减措施的支持度更低。此外, 距离保护区远近与人类足迹指数的影响较弱且不一致, 但随机截距表明各村庄对管理方案的偏好存在显著差异。以上结果表明, 保护策略应优先解决与野生动物相关的成本问题, 并通过更公平地分配收益来促进人兽共存。可能的策略包括将旅游收入投入到共同管理、因地制宜的危害预防措施之中。**【翻译:胡怡思;审校:聂永刚】**

## INTRODUCTION

The co‐occurrence of people and wildlife creates arenas for interactions that can have major consequences for both (Frank et al., 2019). Wildlife species hold ecological, economic, cultural, and spiritual values for human societies (Brauman et al., 2020; Pascual et al., 2017), but they also impose costs (Ceauşu et al., 2019). Wildlife species damage crops and property, prey on livestock, transmit pathogens to livestock, pets, and humans, and injure or kill humans (Kiffner & Ostermann‐Miyashita, 2024; Nyhus, 2016). Concerns over food security, safety, and human well‐being often prompt people to kill wildlife preemptively or in retaliation (Davies et al., 2011; Fitzherbert et al., 2014; Kissui et al., 2022; Nyhus, 2016; Salerno, Andersson, et al., 2021).

To reconcile biodiversity conservation with human interests in shared landscapes, strategies for achieving sustainable human–wildlife coexistence, defined as a dynamic state that ensures wildlife population persistence while reducing wildlife‐related risks to “tolerable levels” (Carter & Linnell, 2016), are needed (Gross et al., 2021; König et al., 2021; Pooley et al., 2020). In general, methods for mitigating wildlife‐related impacts can be categorized as technical measures that protect human assets from wildlife (e.g., fencing or deterrents); interventions that reduce the number of wildlife (e.g., selective or nonselective removal); or financial mechanisms that offset wildlife‐related impacts (e.g., compensation, insurance, or conservation payment schemes) (Conover, 2002; Dickman et al., 2011; Kiffner & Ostermann‐Miyashita, 2024). Taking no action is also an option, whether due to a lack of necessity, resource constraints, or an intentional decision to tolerate some risk engendered by wildlife, where tolerate refers to voluntarily refraining from actions that would harm wildlife despite holding negative or ambivalent attitudes toward it (Lehnen et al., 2022). In principle, all four broad management options can contribute to human–wildlife coexistence, but focusing on reducing wildlife populations may a wildlife persistence (Carter & Linnell, 2023; Ripple et al., 2014, 2016).

Progress has been made in assessing the technical effectiveness of wildlife damage mitigation (Eklund et al., 2017; Killion et al., 2021; Treves et al., 2019; van Eeden et al., 2018), and understanding public perceptions of these methods is crucial for two reasons. First, bottom‐up decision‐making that accounts for the preferences of those directly interacting with wildlife can foster a sense of ownership and legitimacy, which in turn can reduce opposition to management interventions (Hansen et al., 2022; Marino et al., 2021). When technical measures align with preferences of people affected by wildlife, they are more likely to be adopted at scale (Denninger Snyder & Rentsch, 2020; Kiffner et al., 2021; Liordos et al., 2017). Second, so‐called human–wildlife conflicts typically reflect disagreements over goals for wildlife management, such as between farmers and conservationists (IUCN, 2023; Peterson et al., 2010; Redpath et al., 2013). Therefore, understanding local wildlife management preferences can help identify areas of tension and common ground (Zimmermann et al., 2020).

Although research has provided insights on people's values and beliefs (Dietsch et al., 2016; Kioko et al., 2015), attitudes (Kansky & Knight, 2014; Kansky et al., 2014), and tolerance for certain wildlife species (Jacobsen et al., 2020; Kansky et al., 2016, 2021), less attention has been directed toward understanding how specific factors shape preferences for wildlife management options (but see, e.g., Dheer et al. [2021]). This is a crucial missing link. Although attitudes and tolerance can inform broad conservation issues, understanding how people perceive management actions is necessary for implementing strategies that are effective and socially acceptable (Volski et al., 2021). Early work has often relied on the potential for conflict index, which quantifies the level of consensus over management decisions (Manfredo et al., 2003; Vaske et al., 2010). This index has been applied across diverse contexts (e.g., Engel & Vaske, 2022), including mapping support for (or disagreements over) wildlife management strategies (e.g., Engel et al., 2017; Heneghan & Morse, 2019). Although these studies provide valuable insights, they are often descriptive and do not consider why people prefer certain management options over others.

Narrowing this research gap is particularly important in East African savannas, where people live alongside wildlife (Fynn & Bonyongo, 2011; Kiffner, Bond, et al., 2022). Human–wildlife interactions in East African savannas involve multiple species, each presenting distinct interactions, risks, benefits, and management challenges. Because human‐dominated areas are key to the persistence of wide‐ranging species (Bond, Kiffner, et al., 2022; Kiffner et al., 2024; Ogutu et al., 2017) and wildlife can affect people's livelihoods (Kissui et al., 2022; Raycraft, 2023, 2024a), conservationists need to move beyond descriptive or single‐species analyses and identify factors that shape preferences for wildlife management (Jacobs et al., 2018).

One of the most fundamental influences on people's judgments of wildlife is the perceived costs and benefits of living with wildlife. The hazard‐acceptance model (Bruskotter & Wilson, 2014) posits that tolerance for wildlife reflects an internal trade‐off between tangible and intangible costs and benefits. This mechanism is supported by empirical studies of wildlife management preferences (Carter et al., 2012; Zajac et al., 2012). However, although both tangible and intangible costs and benefits are consequential for human well‐being, few studies have examined their relative influence on wildlife management preferences (but see Kansky & Knight [2014] and Jacobsen et al. [2020]).

Beyond perceived costs and benefits, landscape context could mediate wildlife management preferences. Given that human–wildlife interactions occur in coupled social–ecological systems (Lischka et al., 2018), their nature is influenced by the interplay of environmental, ecological, and human factors (Araneda et al., 2022). Hence, landscape factors that influence the distribution of wildlife, human activities, and the frequency of encounters can influence perceptions of cost–benefit trade‐offs (Muneza et al., 2022; Sage et al., 2022; Teixeira et al., 2021). The not‐in‐my‐backyard phenomenon suggests that although people may support wildlife conservation in principle, their tolerance for wildlife often declines with increasing proximity (Zimmermann et al., 2020). At the local scale, residents living close to protected areas or in areas with higher wildlife densities may face a greater risk of negative interactions and thus may be more likely to prefer population size reduction (Koziarski et al., 2016). Similarly, the human footprint index (HFI), an aggregate measure of land‐use intensity (details in Appendix S1), modulates wildlife distributions and human activities. Therefore, the HFI could also influence human–wildlife interactions and cost–benefit perceptions (Hoare & du Toit, 1999; Tucker et al., 2020). However, the direction and strength of the HFI's influence on human perceptions of wildlife management preferences are difficult to predict. On the one hand, in high‐HFI areas, interactions with wildlife may be less frequent but more severe when they do occur. On the other hand, in areas with low HFI, where residents regularly interact with wildlife, tolerance may be higher due to familiarity with mitigation strategies.

To assess the relative strength of perceived costs and benefits and spatial variables (distance to protected area and HFI) in influencing wildlife management preferences, we parameterized a hierarchical Bayesian model with responses from a questionnaire administered to residents of rural, northern Tanzania. For analyses, we considered six herbivorous and six carnivorous mammals. Although explicitly considering local context, we sought to gain insight into the relative strengths of socioecological variables in shaping preferred wildlife management options and find an evidence base for effective wildlife management in a fragmented landscape that supports long‐distance movements of large mammals (Bond et al., 2017; Lohay et al., 2022).

## METHODS

### Study area

**FIGURE 1:**
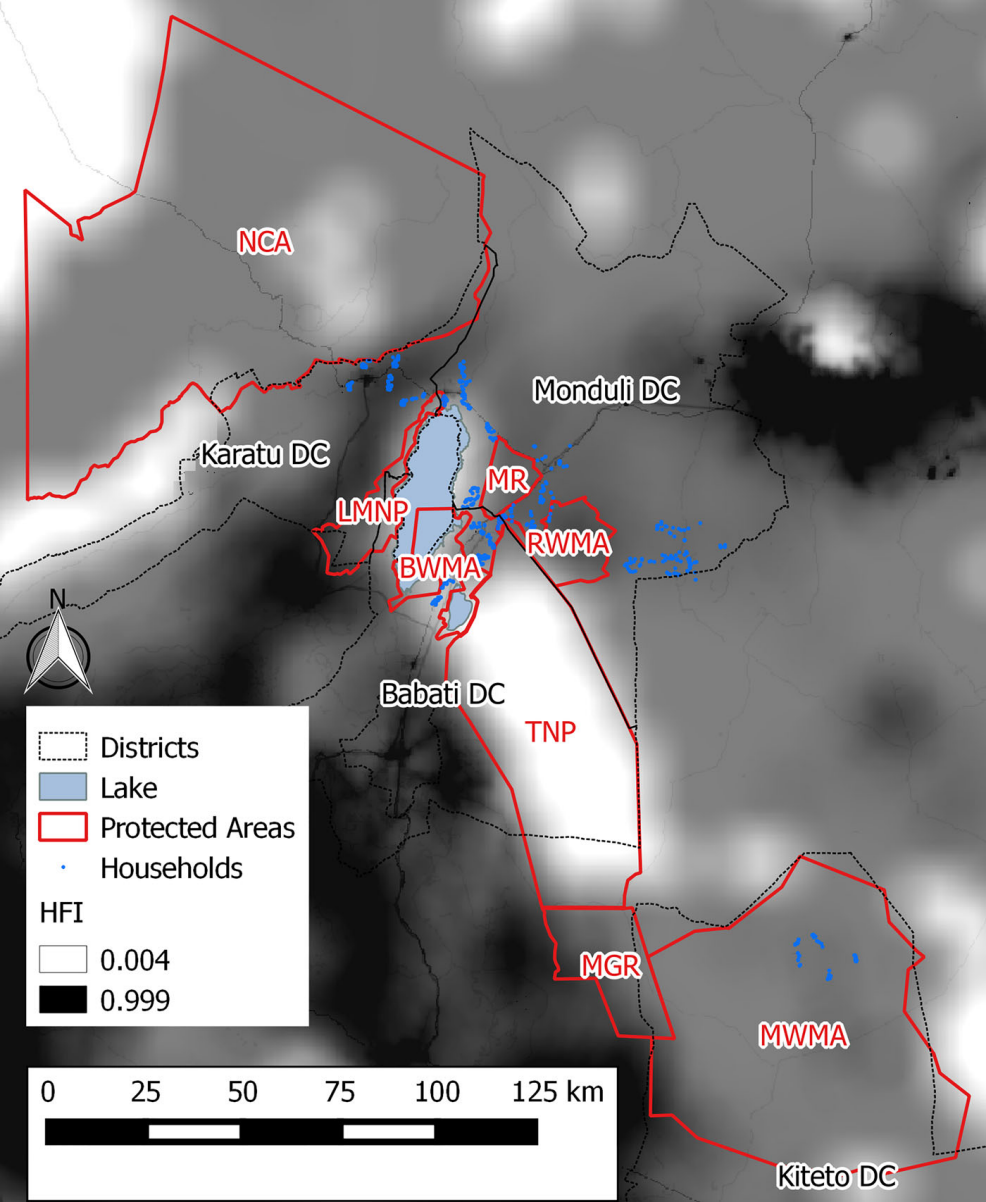
Study area in northern Tanzania; households (blue dots) across four districts (Karatu, Monduli, Babati, Kiteto) where interviews about wildlife management preferences were conducted; key protected areas (BWMA, Burunge Wildlife Management Area; LMNP, Lake Manyara National Park; MGR, Mkungunero Game Reserve; MR, Manyara Ranch; MWMA, Makame Wildlife Management Area; NCA, Ngorongoro Conservation Area; RWMA, Randilen Wildlife Management Area; TNP, Tarangire National Park); alkaline lakes; and the gradient of the human footprint index (HFI) (grayscale background).

We conducted this study in four districts (Babati, Karatu, Kiteto, and Monduli) of northern Tanzania in 25 villages in a well‐established protected area network (Figure 1) that included Ngorongoro Conservation Area, Lake Manyara National Park, Tarangire National Park, Manyara Ranch, Burunge Wildlife Management Area (WMA), Randilen WMA, and Makame WMA. The protected areas in our study area are unfenced and contain species‐rich wildlife assemblages. We focused on six herbivores and six carnivores. The herbivores included African elephant (*Loxodonta africana*), giraffe (*Giraffa camelopardalis tippelskirchi*), buffalo (*Syncerus caffer*), zebra (*Equus quagga*), wildebeest (*Connochaetes taurinus*), and impala (*Aepyceros melampus*). The carnivores included African lion (*Panthera leo*), hyena (*Crocuta crocuta* and *Hyena hyena* combined because respondents frequently did not differentiate between these two species), leopard (*Panthera pardus*), cheetah (*Acinonyx jubatus*), black‐backed jackal (*Lupulella mesomelas*), and honey badger (*Mellivora capensis*). These wildlife species move seasonally across the ecosystem or occupy village lands year‐round (Kiffner et al., 2016) and interact with people in rural areas (Bencin et al., 2016). However, large mammal assemblages differ by location. For example, giraffe, zebra, wildebeest, impala, and cheetah are not present in the Karatu highlands, yet elephants and other species inhabit the area (Diplock et al., 2018; Kiffner et al., 2021).

The human population of the study area is ethnically diverse and includes agropastoralist Iraqwi and WaArusha, predominantly pastoralist Kisongo Maasai, and numerous other ethnic groups. Additional information on the study area and summary statistics of the respondents is in Appendices S1 and S2.

### Questionnaire survey and respondent‐related variables

Before conducting our survey (carried out from 2017 to 2020 [details in Appendix S1]), we obtained permission from the Tanzania Wildlife Research Institute and the Tanzania Commission for Science and Technology (permits 2016‐349‐NA‐2013‐191, 2017‐288‐ER‐2013‐191, 2019‐92‐NA‐2103‐191, 2019‐426‐NA‐2019‐299, CST00000398‐2024‐2024‐00240) and obtained letters of permission from Babati, Karatu, Kiteto, and Monduli district governments. Research was conducted in accordance with human research ethics reviews from McGill University (479–0419) and the University of Alberta (Pro00130079). The survey protocol was reviewed and exempted from further review by the Academic Office at the School for Field Studies. We also obtained permission from leaders in each village.

To capture the diversity of local socioecological contexts, we selected villages based on their spatial distribution across the ecosystem and their distance to protected area boundaries. The sample included villages affiliated with community‐based conservation models, such as WMAs (village representatives manage the area according to a land‐use plan; wildlife‐related revenue is used to manage the area and distributed to member villages) and Manyara Ranch (partner villages receive monetary and in‐kind support and are represented in the governing board of the ranch), and villages not part of such schemes. Although the sampling design captured a broad range of socioeconomic and governance settings, it was not structured as a matched sampling scheme between community‐based and non‐community‐based conservation villages.

To sample respondents from each village, research teams (one or two coauthors and a research assistant or a single research assistant) walked in predetermined directions radiating from the approximate village center and approached spatially separated households, maintaining a minimum distance of ∼200 m between them. Although this sampling approach does not guarantee full representativeness of the rural population, it enabled spatial coverage across core inhabited areas and included an element of randomization.

Upon approaching households, we introduced ourselves, explained the study, guaranteed anonymity, and granted the person the right to cease participation at any time. After participants verbally stated consent to participate, we started the survey with one member (≥18 years of age) of the household. Depending on the respondent's preference, we conducted the survey in *kiSwahili* (the national language of Tanzania) or *Maa* (the language of the Maasai). The first part of the structured questionnaire (Appendix S3) was designed to establish the demographic and socioeconomic background of the respondent. The second part included questions pertaining to the 12 wildlife species. For each species, we assessed whether participants could identify it by showing a picture and asking them to name the species. We coded these responses as either correct (respondent accurately named the species), partial (e.g., respondent provided only a nonspecific name for a species and did not specify further when prompted), or false (e.g., naming a buffalo as a rhinoceros). As the target variable in our analyses, we used a forced‐choice question format. For each of the 12 species, we asked participants to select their preferred wildlife management option: none, prevent damages (prevent), compensate for damages (compensate), or reduce the population size (reduce).

As key variables to explain variation in preferred management options, we considered perceived costs and benefits. For each species, we asked respondents in a structured format whether they had experienced any costs and benefits and, if so, to briefly describe the nature of these (Appendix S3). Although responses were open‐ended, the species‐by‐species structure of the questionnaire encouraged concise answers. After the surveys were completed, we categorized costs and benefits into the following categories: none, tangible (e.g., direct damage to crops or property, livestock predation; direct benefits, such as meat, skin, or body parts), intangible (threats to human health, pathogen transmission, damage to the environment; indirect benefits, such as cultural values, aesthetic values, ecological roles), and multiple (i.e., respondent mentioned intangible and tangible costs or any combination of tangible, intangible, and tourism benefits). We defined tangible impacts as those that are direct and easy to quantify economically; intangible impacts (though potentially consequential) are more difficult to quantify economically. For benefits, we also included “tourism” as a separate category because it was unclear whether respondents perceived it as tangible (i.e., if they directly profited from tourism) or intangible (i.e., if they valued the species for its contribution to tourism in general).

### Spatial variables

To test for generalizable spatial influences on preferred wildlife management options, we considered the HFI and the distance to the nearest protected area (PA distance). We used the HFI dataset by Kennedy et al. (2019), which provides a cumulative, continuous, and recent (median year 2016) measure of human landscape modification based on 13 anthropogenic stressors. The HFI ranges from 0 (no human modification) to 1 (completely modified area). To generate one HFI score for each household, we overlaid the rastered HFI data with the locations of the households in QGIS 2.8.3 and used the geoprocessing tool (Figure 1). Because human activities, such as grazing and crop cultivation, are not spatially limited to the location of the residence (recorded using a handheld GPS), we estimated the average HFI within a 1.94‐km area around each household. This radius represented the median distance of cattle displacement in northern Tanzania (Ekwem et al., 2021). To calculate the distance to the nearest protected area, we used the NNjoin function; for households located within the boundaries of protected areas, we set the distance to 0 km.

### Data analyses

To test how perceived costs and benefits and spatial variables influenced preferred management options for 12 wildlife species, we used a Bayesian hierarchical model implemented in R 4.1.1 (R Core Team, 2021), broadly following Heiss (2023). We ran our analyses with the brms package (Bürkner, 2021). We obtained 680 completed questionnaires. We included only cases in which respondents correctly or partially identified species. Inquiries about the dataset can be directed to the corresponding author.

As fixed effects, we included perceived costs (none, intangible, tangible, multiple) and benefits (none, intangible, tangible, multiple, tourism) associated with each species. In addition, we included the HFI and PA distance and categorized these spatial variables as small, medium, or large. This categorization, based on the quartiles of their distributions, allowed us to capture potential nonlinear effects on preferences for management options.

To account for the nested data structure, we included random effects for species and for respondents nested within villages. Nesting respondents within villages accounted for the possibility that individual responses may be influenced by shared local context (Dickman et al., 2014). The respondent‐level random effect captured repeated measures as respondents provided answers for all species. The species‐level random effect accounted for systematic differences in preferred management options across wildlife species, regardless of fixed effects.

We specified the model as a categorical outcome with a multinomial distribution and logit link function and suppressed the intercept to ensure that the none option served as the baseline. We fitted the model with four Markov chain Monte Carlo chains, each with 8000 iterations, including a 4000‐iteration warm‐up period. To improve model convergence, we set the adapt‐delta parameter to 0.98 and the maximum tree depth to 14. We checked model convergence with standard diagnostics and *R*‐hat values were consistently equal to 1.00.

To visualize the fixed effects, we used two complementary approaches. First, we computed and plotted the marginal means of each management option across the different levels of the fixed effects. Using the marginaleffects package (Arel‐Bundock et al., 2024), we estimated marginal effects as average predicted probabilities of selecting each management option, along with their credible intervals, given the levels of the fixed effects. In the second approach, we conducted pairwise comparisons between the reduce management option and the other options (none, compensate, and prevent). We used the tidybayes package (Kay & Mastny, 2024) to extract the posterior distributions of the predicted probabilities for each management option and to calculate the differences between these probabilities. This allowed us to quantify the relative impact of the spatial and perceptual factors on the preferences for the reduce management option relative to other options. Finally, we extracted and visualized the random effects for species and villages.

## RESULTS

### Preferred wildlife management options

**FIGURE 2:**
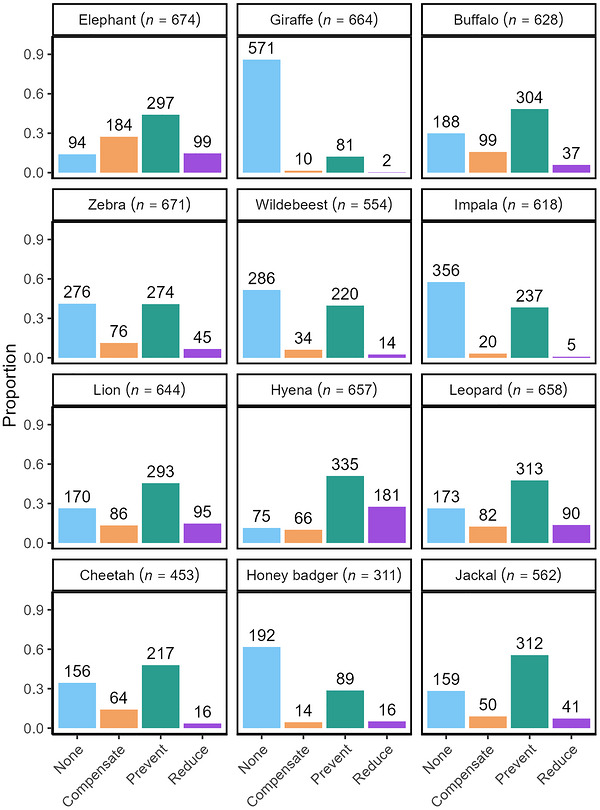
Proportions of preferred management options for six herbivore and six carnivore species based on responses from rural residents of northern Tanzania (reduce, reduce population size; *n*, number of valid responses [i.e., whether the respondent was able to at least partially identify the species]; numbers above bars, number of responses per management option).

Across all species, the majority of respondents preferred either the prevent (41.9%) or none (38.0%) option. The compensate (11.1%) and reduce (9.0%) options were less preferred. A larger proportion of respondents preferred the reduce option for carnivores (13.4%) compared with herbivores (5.3%). At the species level, the preferences varied considerably (Figure 2). Among herbivores, respondents most commonly preferred reduce for elephant (14.7%), zebra (6.7%), and buffalo (5.9%). Among carnivores, respondents particularly favored reduce for hyena (27.5%), lion (14.8%), and leopard (13.7%).

### Predicting preferred wildlife management options

**FIGURE 3:**
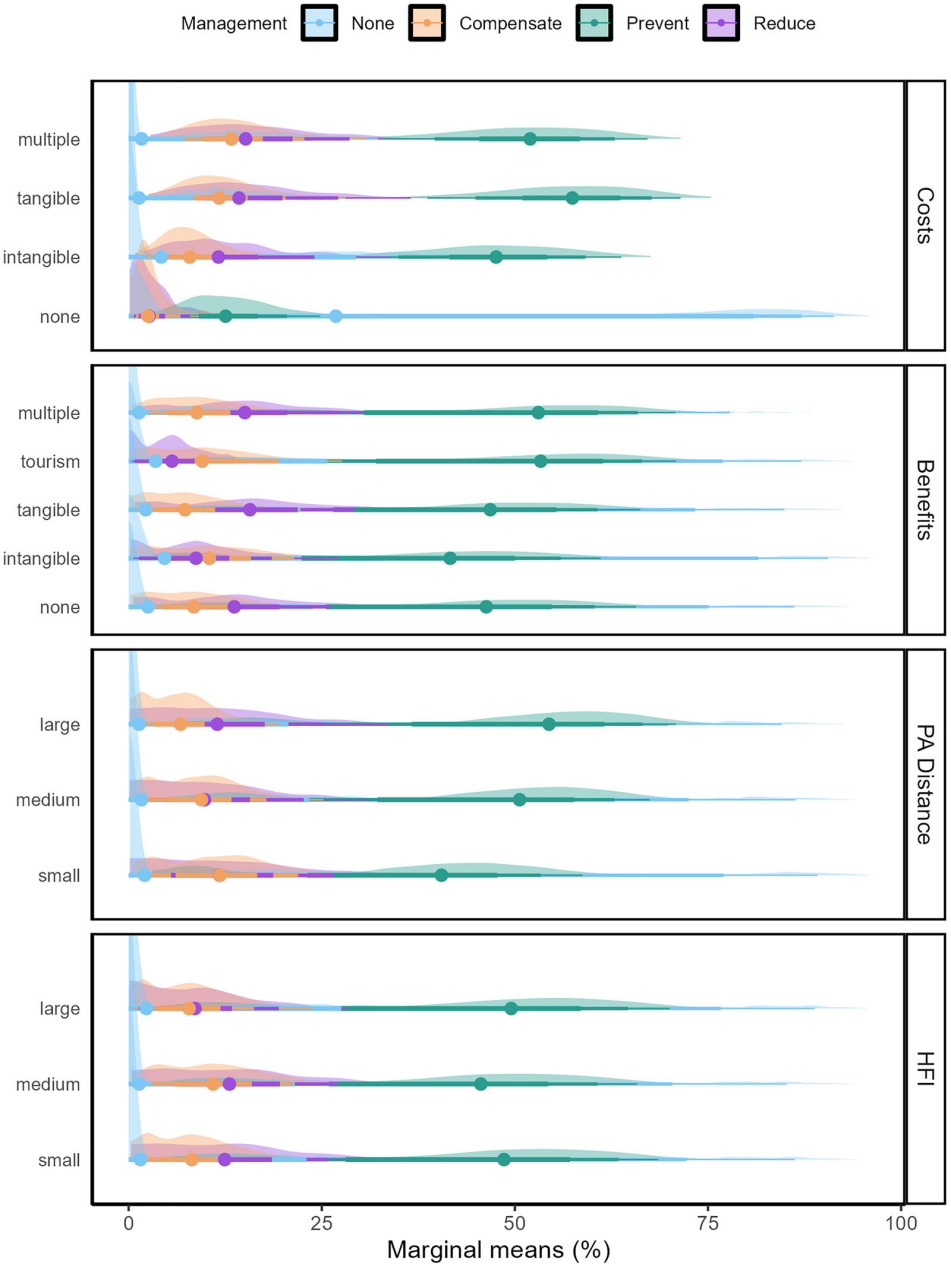
Marginal effects of distance from protected area (PA) (distance, three levels), human footprint index (HFI) (three levels), and perceived costs (four levels) and benefits (five levels) on preferred wildlife management options (compensate, prevent, reduce population size; no management as the reference level) based on a survey of residents of rural, northern Tanzania (density ridges and point intervals, predicted probabilities of selecting each option across the different levels of the explanatory variables). These predictions are based on a Bayesian multilevel model with random effects for respondents, species, and villages.

The Bayesian model revealed that perceived costs had the strongest and most consistent effect on preferred management options (Appendix S4). Respondents who perceived tangible or multiple costs tended to select prevent (posterior mean for tangible costs: 0.57 [95% credible interval 0.00 to 0.99]; multiple costs: 0.52 [0.00 to 0.99]). Conversely, respondents who did not perceive costs predominantly preferred none (0.40 [0 to 1], likely reflecting uncertainty near the boundary of the probability scale) (Appendix S4; Figure 3).

Perceived benefits had a weaker and less consistent effect on management preferences. When respondents perceived multiple or tangible benefits, the probability for selecting reduce was modest (posterior mean for multiple benefits: 0.16 [95% credible interval 0.00 to 0.89]; tangible: 0.16 [0.00 to 0.87]) (Appendix S4). By contrast, respondents who associated wildlife species with intangible or tourism‐related benefits were less likely to choose reduce (intangible: 0.10 [0.00 to 0.74]; tourism: 0.06 [0.00 to 0.63]) (Appendix S4; Figure 3).

The spatial variables had weak effects on preferred management options. Yet, there was support for an effect of distance to protected areas and the preference for prevent. Respondents located farther from protected areas were more likely to prefer prevent at large (0.48 [95% credible interval 0.00 to 0.97]) and medium distances (0.44 [0.00 to 0.95]), compared with those in the small distance category (0.36 [0.00 to 0.92]) (Appendix S4). However, the posterior distributions for reduce were similar across distance categories (small: 0.13 [0.00 to 0.79]; medium: 0.11 [0.00 to 0.78]; large: 0.13 [0.00 to 0.82]), indicating no clear effect (Appendix S4; Figure 3).

The relationship between the HFI and preferred management options showed no clear pattern. The posterior mean probability of selecting reduce was similar across HFI levels, with a slight tendency toward lower probabilities at large HFI levels (small: 0.14 [95% credible interval 0.00 to 0.82]; medium: 0.14 [0.00 to 0.83]; large: 0.10 [0.00 to 0.64]), but the credible intervals suggest this was a weak trend (Appendix S4; Figure 3).

### Pairwise comparisons between reduce and other management options

**FIGURE 4:**
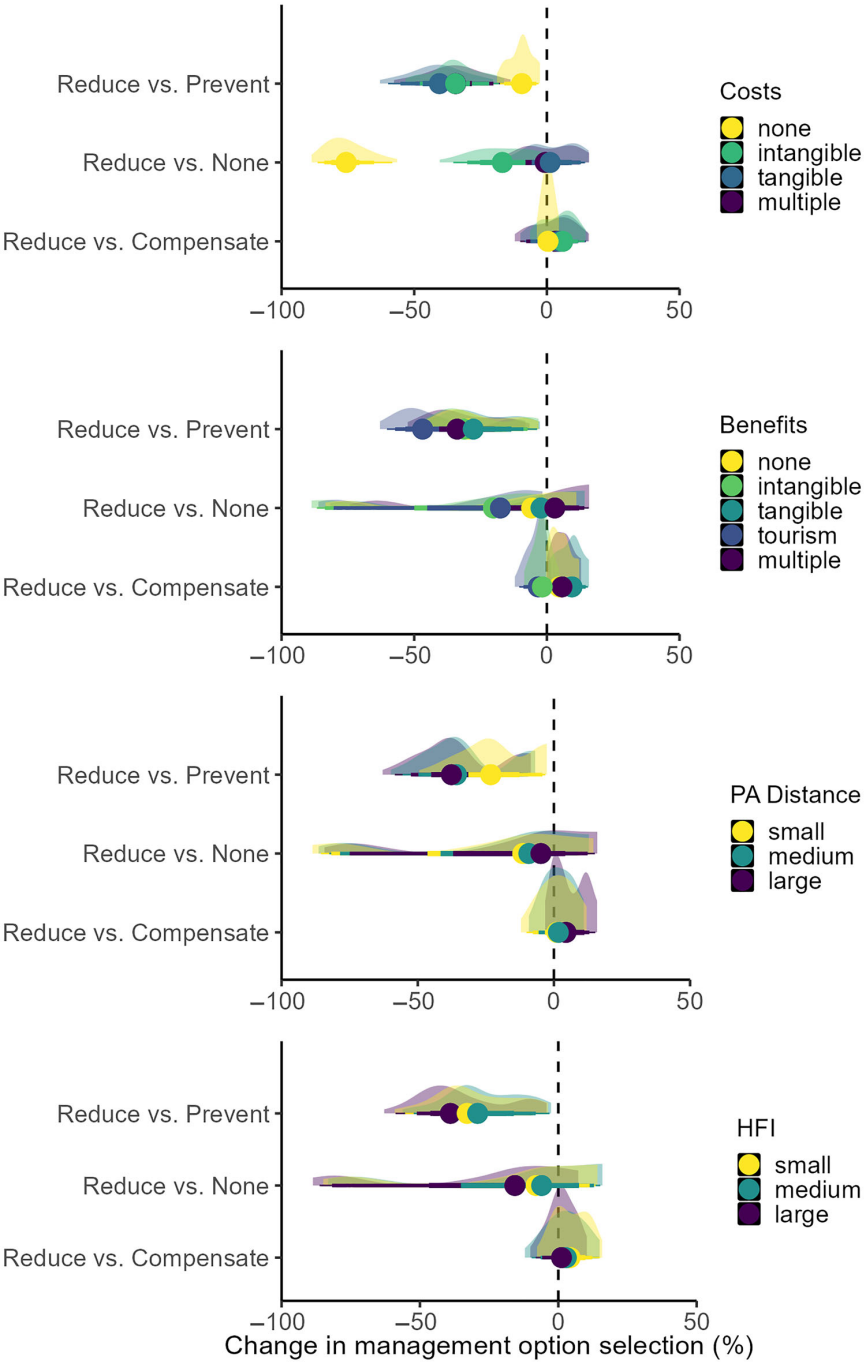
Median (95% credible intervals) predicted differences in the selection probabilities of wildlife management options by survey respondents in rural, northern Tanzania relative to the reduce the population size option and other options (compensate, prevent, or no management [none]) across different levels of explanatory variables: distance to protected area (PA distance), human footprint index (HFI), and perceived costs and benefits associated with wildlife species. Predictions are derived from a Bayesian multilevel model with random effects for respondents, species, and villages to account for variability across these groups.

Pairwise comparisons between the reduce and other management options (none, prevent, and compensate) revealed clearer trends for perceived costs and weaker or inconsistent effects for perceived benefits and spatial variables (Figure 4). Although all credible intervals included zero, these differences reflected the range of plausible values for these differences given the data.

Perceived costs had the strongest effect across pairwise comparisons. Respondents who did not perceive any costs were more likely to prefer none over reduce (mean difference: −0.83 [95% credible interval −1.00 to −0.17]). When respondents reported tangible or multiple costs, the likelihood of choosing reduce over compensate was similar (mean differences: multiple = 0.02 [−0.96 to 0.94]; tangible: 0.03 [−0.94 to 0.94]), suggesting no strong preference for one over the other in such cases. Even when tangible costs were perceived, respondents tended to favor prevent over reduce (mean difference for prevent vs. reduce: tangible −0.49 [−0.96 to 0.92]; multiple −0.40 [−0.93 to 0.92]).

Perceived benefits showed mixed and weak effects. Respondents were slightly more likely to select reduce over compensate when they perceived tangible benefits (0.09 [95% credible interval −0.44 to 0.82]). However, perceived tourism benefits were associated with a shift away from reduce toward none (−0.33 [−0.90 to 0.38]) and prevent (−0.45 [−0.90 to 0.53]). Similarly, distance to protected area and levels of HFI did not meaningfully distinguish preferences for reduce over other management options.

### Random effects

**FIGURE 5:**
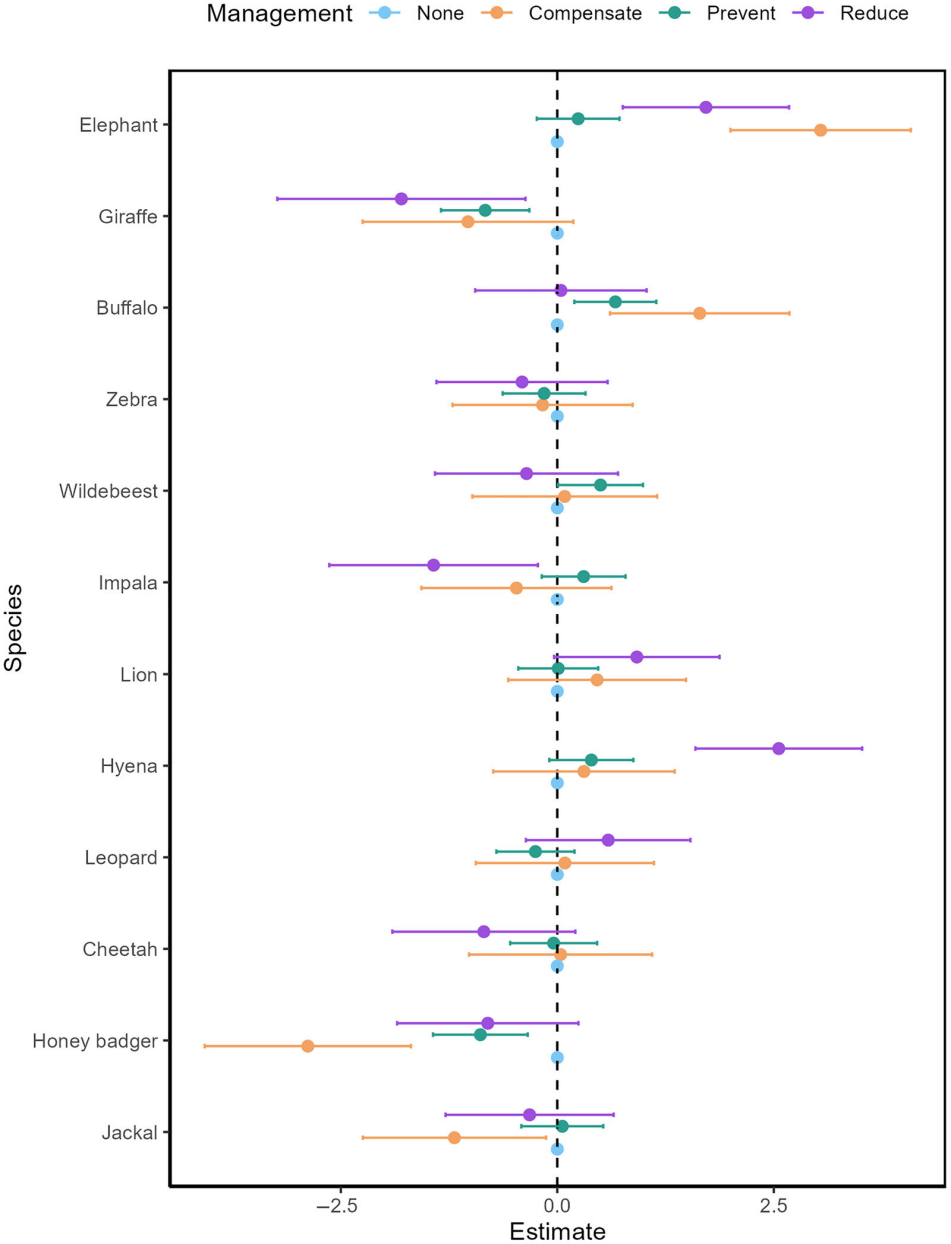
Species‐level random effect estimates from a Bayesian multilevel model predicting the wildlife management preferences of rural residents in northern Tanzania (points, estimated random effect size for a species on the preferred wildlife management options [compensate, prevent, reduce population size]; none [i.e., no management], reference category; error bars, 95% credible intervals).

**FIGURE 6:**
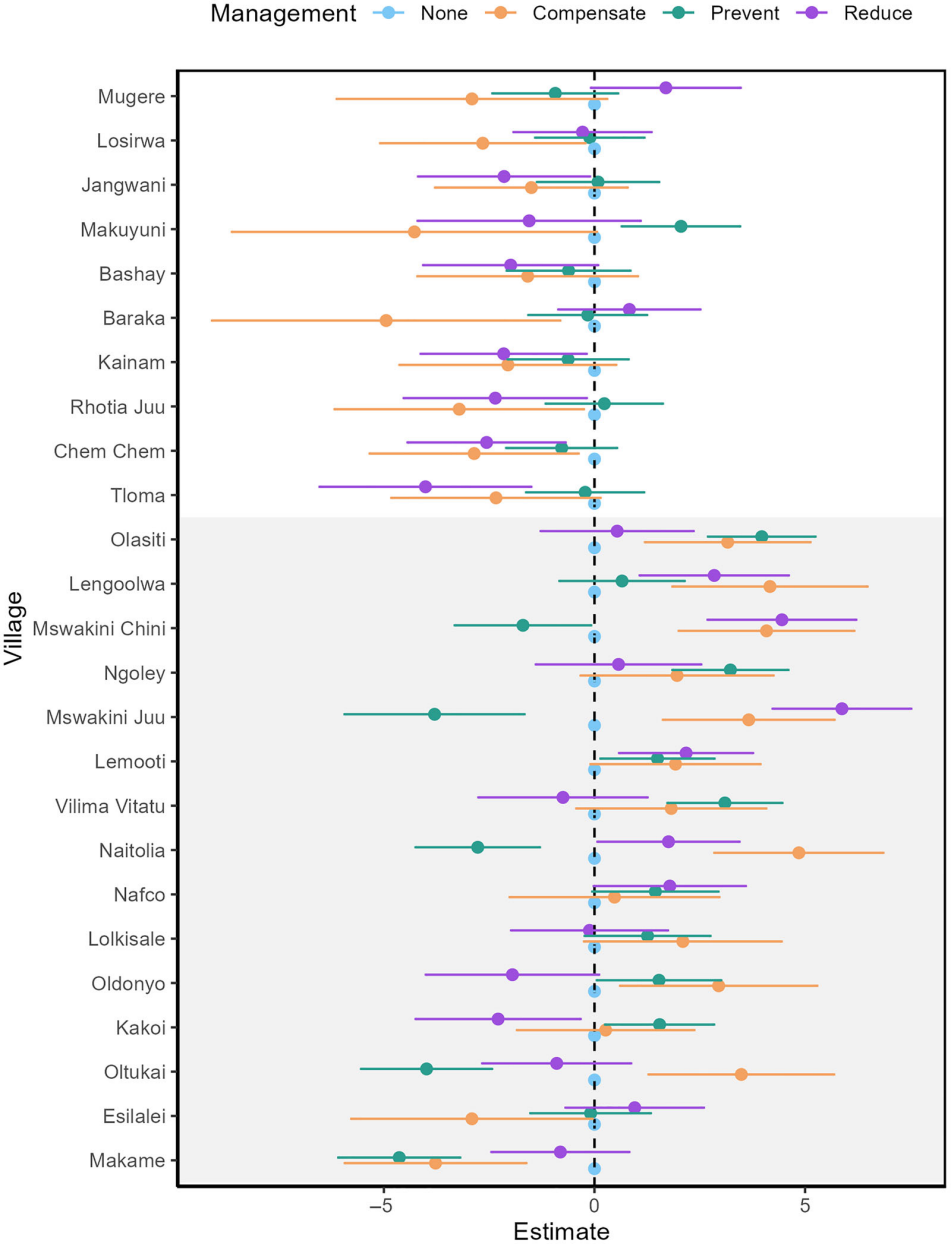
Village‐level random effect estimates from a Bayesian multilevel model predicting the wildlife management preferences of rural residents in northern Tanzania (points, estimated random effect size for a village for one of the three preferred wildlife management options [compensate, prevent, reduce population size]; none [i.e., no management], reference category; error bars, 95% credible intervals; shading, villages that are part of community‐based conservation models).

Variation at the respondent‐within‐village level was substantial, with SD 2.75 (95% credible interval 2.38 to 3.15) for compensate, 1.81 (1.62 to 2.01) for prevent, and 2.05 (1.74 to 2.39) for reduce. Species‐level random effects revealed major variation in preferences for compensate (SD 1.62 [1.04 to 2.54]), prevent (SD 0.58 [0.34 to 0.98]), and reduce (SD 1.44 [0.90 to 2.34]) (Figure 5). Support for compensate was strongest for elephant (SD 3.04 [2.00 to 4.09]) and buffalo (SD 1.64 [0.61 to 2.70]), and lowest for honey badger (SD −2.88 [−4.13 to −1.73]) and jackal (SD −1.19 [−2.29 to −0.14]). For prevent, wildebeest (SD 0.50 [0.02 to 1.00]) and buffalo (SD 0.67 [0.20 to 1.16]) had the highest estimates, whereas honey badger (SD −0.89 [−1.46 to −0.37]) and giraffe (SD −0.83 [−1.37 to −0.34]) had the lowest. Preferences for reduce were highest for hyena (SD 2.56 [1.62 to 3.56]) and elephant (SD 1.72 [0.76 to 2.70]), and lowest for giraffe (SD −1.80 [−3.38 to −0.47]) and impala (SD −1.43 [−2.70 to −0.27]) (Figure 5). The village‐level random intercepts exhibited high variability, particularly for compensate (SD 3.36 [2.39 to 4.68]). Prevent (SD 2.35 [1.73 to 3.21) and reduce (SD 2.57 [1.84 to 3.58]) showed similarly large variations across villages. Villages associated with community‐based conservation models generally exhibited higher estimates for compensate and reduce than villages not associated with community‐based conservation (Figure 6).

## DISCUSSION

Based on a large sample from an ethnically and environmentally diverse setting in northern Tanzania, our fitted hierarchical Bayesian model suggested that perceived costs were the strongest predictor of preferred management options for large mammal species. Perceived benefits and spatial variables had weaker and inconsistent effects. However, associating wildlife species with tourism or intangible benefits was linked to lower support for wildlife population reductions.

### Relative importance of costs and benefits

Our findings support the hazard‐acceptance model in the broad sense that perceived costs and benefits are key determinants of individual preferences regarding contentious issues, such as wildlife management (Bruskotter & Wilson, 2014; Siegrist et al., 2000). However, unlike findings from a study on preferred population sizes of tigers (*Panthera tigris*) in Nepal that identified perceived benefits as the strongest predictor (Carter et al., 2012), our results suggested that perceived costs were more influential in shaping preferences for wildlife management in northern Tanzania. Specifically, multiple perceived costs, followed by tangible and intangible costs, were strongly associated with a greater likelihood of preferring population reduction over nonlethal alternatives (Figures 3 & 4).

The greater relative importance of tangible over intangible costs in our study contrasts with findings from a study on attitudes toward lions in Zimbabwe, where intangible factors were as important, if not more important, than tangible costs, such as livestock loss (Jacobsen et al., 2020). Similarly, a meta‐analysis showed that intangible costs were the most consistent predictors of attitudes toward wildlife across species and contexts, whereas tangible costs and benefits were often not strongly associated with attitudes toward wildlife (Kansky et al., 2014). A recent study also showed that intangible benefits and emotions best predicted tolerance toward wildlife, whereas monetary benefits had limited effects (Kansky & Kidd, 2024).

Our finding that perceived tangible benefits were generally less influential than costs in predicting preferred management aligns with Kansky and Knight (2014). In our study area, tangible wildlife‐related benefits are few. Consumptive use of wildlife is largely illegal (Kiffner et al., 2015), and revenues generated through entrance and concession fees of protected areas often fund communal education and health infrastructure rather than offset the household‐level costs of living with wildlife (Kegamba et al., 2022). Even where villages are part of WMAs (which are designed to generate income through wildlife‐based tourism [Wilfred, 2010]), their impact on household income is small and difficult to attribute directly to WMA activities (Kegamba et al., 2022). Moreover, these benefits are unevenly distributed and not tied to wildlife‐related impacts or ecological performance (Homewood et al., 2022; Keane et al., 2020).

Nonetheless, our results suggest that tourism and intangible benefits can influence wildlife management preferences. Respondents who reported tourism‐related benefits were less likely to support population reduction (Figure 3). Similarly, intangible benefits also reduced support for population reduction. This resonates with evidence from the Kavango–Zambezi Transfrontier Conservation Area, where nonmonetary benefits have been associated with greater tolerance for wildlife species (Kansky et al., 2021). These two findings offer clear pathways for building broader support for coexistence. First, a more direct, transparent distribution of tourism‐related income to households bearing the costs of living with wildlife could increase perceived fairness and strengthen local support for nonlethal management. Second, expanding ongoing efforts to raise awareness of intangible benefits through education, outreach, and experiential learning (Bond, Barisha, et al., 2022) can help foster positive associations with wildlife and strengthen support for coexistence‐oriented management.

Although these specific results offer actionable insights, broad conclusions on the relative importance of tangible and intangible costs versus benefits are complicated by challenges in measuring key variables. Although our outcome—preferred wildlife management options—captures an intended behavioral response, it is not directly comparable to attitudinal constructs, normative beliefs (Jacobsen et al., 2020; Kansky & Knight, 2014), or preferred population sizes (Carter et al., 2012). Accordingly, comparisons across studies with different methodologies should be done cautiously. For example, although attitudes often precede behavioral intentions, individuals may express neutral or even positive attitudes toward wildlife but nevertheless support lethal management when facing negative impacts (Rastgoo et al., 2025).

We focused on preferences for general wildlife management options; however, population reduction is not a legal management scenario in Tanzania. Killing an animal in defense of human life or livestock is permitted under strict protocols (The United Republic of Tanzania, 2022), and authorities occasionally shoot or relocate individual animals, but population‐wide control is not officially sanctioned. Nevertheless, we included this option because it does occur illegally (Kissui et al., 2022) and serves as a proxy for intolerance. To shed further light on the extent of illegal wildlife killing, future research should investigate this issue more directly, ideally through specialized questioning techniques that protect respondents’ privacy and encourage more honest responses (Cerri et al., 2021).

The lack of standardized, monetary valuation of costs and benefits (though possible, e.g., Jacobsen et al. [2022]) and not prompting respondents for indirect impacts may have contributed to differences in the relative importance of tangible and intangible costs and benefits in our study. We deliberately avoided asking respondents to quantify wildlife‐related damages because self‐reported figures are often exaggerated (Kissui et al., 2022). Instead, our approach allowed respondents to associate species with costs and benefits (Appendix S3). Perhaps, this method primarily prompted reporting of direct costs, such as crop damage and livestock losses, whereas indirect effects on well‐being (i.e., “hidden impacts” [Barua et al., 2013]) may have been underreported. Indeed, disrupted sleep patterns due to night‐time guarding of crops or livestock and a sense of insecurity have been reported during in‐depth interviews and targeted questionnaires (Raycraft, 2023; Raycraft & Bell, 2025), yet such intangible impacts were rarely mentioned in our questionnaire.

We did not include emotional responses, such as fear, as explanatory variables in our model. Although emotions can shape attitudes and behavioral intentions (Jacobs, 2012; Dheer et al., 2021), a model that included fear failed to converge. Nevertheless, fear likely played a role in shaping management preferences. More than 40% of respondents expressed fear toward 6 of the 12 species (elephant, buffalo, lion, leopard, cheetah, and hyena) (Appendix S5). Population reduction was frequently preferred for these species (Figures 2 & 5). Fear is an intangible cost with profound implications. Feelings of vulnerability, lack of control, and anxiety can persist regardless of whether physical harm exists. In some circumstances, people's fears are grounded in real dangers. Attacks by large herbivores and carnivores occur in northern Tanzania and are sometimes fatal (Kissui et al., 2022; Koziarski et al., 2016; Raycraft, 2023). Stories of such tragic incidents spread within and across communities, potentially amplifying risk perceptions (Dickman et al., 2014).

Although these methodological concerns are important, we hypothesize that our results generally reflect the subsistence‐oriented livelihoods of the studied human population, where wildlife‐caused losses of crops or livestock, in addition to hardships caused by climate variability, crop pests, and livestock diseases, can have immediate and severe consequences for household food security, income stability, and social status (Kissui, 2008; Kissui et al., 2019; Salerno et al., 2016; Salerno, Stevens, et al., 2021). In this context, wildlife‐related risks are not abstract or minor concerns; rather, they are daily realities that directly affect well‐being and livelihoods (Raycraft, 2024a). For pastoralist groups, such as the Maasai, livestock is central not only to livelihoods but also to their cultural identity and social status (Bell et al., 2025; Hampson et al., 2015; Reid, 2012). Similarly, many people in the ecosystem rely on small‐scale, rain‐fed agriculture for income and food security (Kiffner et al., 2021). Losses caused by wildlife, especially when frequent, severe, or occurring at vulnerable times, are particularly salient and pose a threat to overall livelihood security (Salerno, Stevens, et al., 2021).

Overall, these considerations suggest that livelihood security and safety are central concerns for rural residents in northern Tanzania (McCabe, 1997; Raycraft, 2023, 2024a). The cumulative burden of tangible and intangible costs likely explains why such factors emerged as the strongest predictors for supporting wildlife population reductions. This is consistent with psychological theory. Particularly under conditions of resource scarcity, people are more motivated to avoid losses than to pursue gains (Kahneman & Tversky, 1979), and negative experiences tend to be more influential for decision‐making than positive ones (Kansky & Knight, 2014). In such contexts, the stakes of loss are too immediate to be outweighed by indirect or collective benefits alone. Yet, our results highlight a clear leverage point for facilitating human–wildlife coexistence: when people perceive nonmonetary or tourism‐related benefits, they are more inclined to support nonlethal wildlife management.

### Limited evidence for generalizable spatial variables

Although landscape context has been proposed to mediate wildlife‐related perceptions, attitudes, and preferences (Lischka et al., 2018; Ostermann‐Miyashita et al., 2023; Teixeira et al., 2021), the spatial variables we considered had weak and inconsistent effects on preferred wildlife management options. Perhaps, the limited effect of the HFI variable reflects the design of our study that intentionally targeted rural settings and excluded urban centers. As a result, HFI scores in our sample (range 0.32–0.85) do not entail the full rural–urban gradient (0–1). The commonly cited not‐in‐my‐backyard dynamic typically unfolds in studies spanning the full gradient, where urban residents (who are typically distant from wildlife impacts) primarily see wildlife more favorably, whereas rural residents who directly bear the costs of wildlife presence may hold more negative attitudes (Ostermann‐Miyashita et al., 2023; Zimmermann et al., 2020).

At first glance, our findings contrast with those of Muneza et al. (2022), who found that attitudes toward wildlife in Kenya's Tsavo ecosystem were influenced by landscape condition and prior negative experiences and that there was stronger support for population reduction in areas with frequent human–wildlife interactions. Although we did not predict high‐ and low‐risk areas of human–wildlife interactions, our model included perceived costs and benefits, which likely captured much of the variation that spatial variables might otherwise explain. Our model also indicated substantial variation among villages (Appendix S4), suggesting that site‐specific factors play an important role in influencing management preferences. This aligns with studies showing that attitudes and preferences can be influenced by other people's norms and views (Dickman, 2010; Dickman et al., 2014) and are shaped by local contexts (Ruppert et al., 2022).

An alternative explanation is that the chosen spatial variables are poor proxies for human–wildlife interactions in this ecosystem. Wildlife moves widely across the landscape, especially during the wet season (Kiffner, Foley, et al., 2022; Lohay et al., 2022; Morrison & Bolger, 2012), potentially resulting in encounters anywhere and at different times of the year (Mills et al., 2024). Indeed, human–wildlife interactions on village lands, such as livestock predation, are difficult to predict based on spatial features (Hoffmann et al., 2019; Kissui et al., 2019; but see Beattie et al., 2020).

Spatial metrics did not strongly influence management preferences. Instead, perceptions of costs and benefits and the socioecological specificity of human–wildlife interactions were more informative. For instance, respondents from villages affiliated with community‐based conservation models tended to prefer more active management options (Figure 6), a pattern that possibly reflects higher frequencies of human–wildlife interactions in those areas.

### Coexisting with wildlife

In line with other studies from northern Tanzania (Bencin et al., 2016; Dheer et al., 2021), most respondents did not support lethal control of wildlife (Figures 2 & 3), suggesting a remarkably high level of tolerance for wildlife overall. However, even though only a small fraction of respondents preferred population size reductions, this minority could exert a disproportionate influence on public discourse and wildlife populations (Naughton et al., 1999; Ohrens et al., 2016). Retaliatory killings of lions and spotted hyenas continue to occur (Kiffner, Foley, et al., 2022; Kissui et al., 2022; Raycraft, 2024b). Although often prompted by severe real and perceived impacts, these activities may also be fueled by perceptions that authorities are unresponsive to people's concerns about wildlife.

Our results indicated that large carnivores and elephants were frequently associated with high perceived costs (Figures 2 & 5) and fear (Appendix S5), and respondents frequently preferred population size reduction of these species. These risk perceptions are consistent with the species’ involvement in severe and consequential interactions, including livestock predation, crop damage, and human injuries and fatalities (Kiffner, Foley, et al., 2022; Kioko et al., 2022; Kissui et al., 2019; Raycraft, 2023, 2024b). Given that three of these species are of global conservation concern (lion and leopard, International Union for Conservation of Nature Red List status vulnerable [Nicholson et al., 2024; Stein et al., 2025]; elephant status endangered [Gobush et al., 2022]) and that they pose real risks to human safety and livelihoods, effectively managing interactions involving these species is of critical importance.

Coexistence challenges in this ecosystem are not limited to high‐profile species. For example, wildebeest leave protected areas during the wet season to give birth in nutrient‐rich village lands. During this period, they shed alcelaphine herpesvirus 1 (AlHV‐1), the pathogen causing malignant catarrhal fever. In cattle, this disease is highly lethal and can cause severe economic loss (Lankester et al., 2015). In response, herders temporarily avoid grazing in wildebeest calving areas, an example of a culturally embedded, locally driven strategy to mitigate wildlife‐related risk. Despite the substantial concern associated with wildebeest, the vast majority of respondents favored preventative approaches over population reduction (Figures 2 & 3). This pattern underscores that even under high‐cost scenarios, many residents preferred nonlethal responses and sought locally grounded ways to coexist with wildlife.

### Aligning policies and governance to promote human–wildlife coexistence

Although the wildebeest example shows that communities have agency in managing risks through locally embedded, culturally informed coexistence strategies, effectively managing human–wildlife interactions requires more than local initiative. It demands context‐sensitive policy and governance systems that are responsive to local realities and provide sustained support to the people who live with wildlife (Carter & Linnell, 2023; Treves et al., 2009).

Despite this need, governance gaps are evident. In practice, no single measure can fully mitigate wildlife impact. Enforced livestock enclosures have reduced carnivore‐related livestock losses (Grau et al., 2025; Kissui et al., 2019; Lichtenfeld et al., 2015) and deterred carnivore visits to homesteads (Bell & Raycraft, 2025). However, they cannot prevent livestock predation entirely. In theory, financial mechanisms could help absorb residual impacts (Dickman et al., 2011; Ravenelle & Nyhus, 2017). Unfortunately, the Tanzanian consolation payment scheme, intended to alleviate wildlife impacts under specific circumstances, is rarely implemented and characterized by bureaucratic hurdles, delays, and marginal payouts. Furthermore, under the Wildlife Conservation Act, only damages caused by “dangerous animals” (e.g., elephants, lions, hyenas) are eligible (The United Republic of Tanzania, 2022); this limitation is widely known and may contribute to local frustrations.

In response to these institutional shortcomings, local entities and nongovernmental organizations (NGOs) have implemented several solutions. For example, NGOs have provided technical and financial support to enforce livestock enclosures (Kissui et al., 2019; Lichtenfeld et al., 2015). Ranger‐led interventions in Manyara Ranch, conducted in collaboration with the Tarangire Lion Project, have successfully halted lion hunts (Beattie et al., 2020). In some WMAs, like Randilen, village game scouts actively assist farmers in protecting crops. Although these comanagement approaches are effective and merit broader replication, we argue that these need to be mainstreamed and institutionalized to ensure long‐term sustainability and equitable coverage across the ecosystem.

Wildlife Management Areas could offer a potential platform for formalizing governance support for coexistence (Raycraft, 2025). Although WMAs in our study area are improving their capacity to manage human–wildlife interactions, we argue that this localized approach is currently limited in scope. Community‐based conservation models, such as WMAs, primarily generate income from site‐based wildlife‐related tourism, but these revenues are typically insufficient to offset the costs of living with wildlife (Keane et al., 2020). At the same time, WMAs and other village lands maintain wildlife corridors that sustain the migratory wildebeest and zebra populations of Tarangire National Park (Kiffner et al., 2024). Despite their ecological importance, only a small portion of these lands receive direct financial support (Nelson et al., 2010), and tourism operators and the central government (via the national parks system) benefit from revenues associated with these migratory wildlife populations. Addressing this discrepancy in the distribution of monetary costs and benefits associated with mobile wildlife is essential. Without formal governance structures that recognize and reward the stewardship of wildlife on village lands, the persistence of wildlife populations will remain precariously dependent on the goodwill of rural communities.

A practical solution could be to allocate a portion of centrally collected tourism revenue toward establishing coexistence support units. These government‐sanctioned entities could not only serve as rapid response teams to address human–wildlife interactions but also facilitate the codesign and co‐implementation of locally appropriate and effective prevention strategies at scale (Carter et al., 2021). Such an initiative could help ensure that people's concerns about the threats posed by wildlife are represented by public policy and that the revenues generated from wildlife tourism are invested in management practices that directly improve people's lived experiences of well‐being.

### Implications for human–wildlife coexistence

We provided a theory‐grounded framework that integrates responses from a questionnaire survey with remotely sensed data to predict preferred wildlife management options for a diverse suite of large mammal species. We had two key findings: First, perceived costs predominantly influenced management preferences. Reducing negative human–wildlife interactions, through investing in effective, sustainable, scalable, and socially acceptable prevention methods (Denninger Snyder & Rentsch, 2020), is likely the single most important step toward improving coexistence in this region. Second, intangible and tourism‐related benefits were associated with more coexistence‐oriented wildlife management options. This suggests that equitably distributing tourism‐related benefits among rural communities, for example, by reinvesting some tourism revenues into comanagement structures and fostering awareness of intangible values through culturally sensitive environmental education (Bond, Barisha, et al., 2022), may strengthen support for human–wildlife coexistence. Although grounded in the social–ecological realities of northern Tanzania, this analytical framework is broadly applicable and can be adapted to inform human–wildlife coexistence strategies on shared landscapes worldwide.

## Supporting information



Supporting information
