# Supplementary material for: Perceived costs as drivers of wildlife management preferences in rural Tanzanian communities
Source: Conserv Biol. 2026 Mar 9;40(4):e70251. doi: 10.1111/cobi.70251 (PMC13392751; doi:10.1111/cobi.70251)
Supplement: Supplementary file 1 — Supporting information [file COBI-40-e70251-s001.docx]

# Supporting information for

# “Perceived costs as drivers of wildlife management preferences in rural Tanzanian communities”

# Appendix S1

## Study area

The 25 surveyed villages are located in four districts (Babati, Karatu, Kiteto, and Monduli) of northern Tanzania. Villages were primarily selected based on their proximity to protected areas and we sought to include villages associated with community-based conservation models and villages not associated with such entities.

The northwestern part of our study area (i.e. selected villages within Karatu District located in the highlands at elevation between 1300-1700 m asl.), is adjacent to the northern highland forest reserve that is part of the Ngorongoro Conservation Area (NCA) (Homewood & Rodgers 2004). People residing in this area mostly belong to the Iraqwi ethnicity (Appendix S2) and carry out small-scale, subsistence farming on the relatively fertile soils of the Karatu highlands (mostly cultivating maize, beans and pigeon peas). Annual precipitation in the highlands is typically around 1000 mm (Prins & Loth 1988). The wildlife assemblage in the Karatu highlands is relatively impoverished and savannah species such as giraffe, zebra, wildebeest, impala, and cheetah are missing (Diplock et al. 2018). However, the adjacent forest of the NCA contains populations of elephant (*Loxodonta africana*), buffalo (*Syncerus caffer*) and multiple carnivore species including the occasional occurrence of lions (*Panthera leo*) (Homewood & Rodgers 2004).

Monduli and Babati districts are located in the central part of the Tarangire Ecosystem. Village lands in Monduli and Babati districts, at elevations between 980-1200 m asl., typically receive less rainfall than areas located in the Karatu highlands (Prins & Loth 1988). Villages sampled in Monduli and Babati districts are located between Lake Manyara National Park (LMNP) and Tarangire National Park (TNP). Adjacent to these national parks, several community-based conservation areas have been established: Burunge Wildlife Management Area (BWMA), Randilen Wildlife Management Area (RWMA), and Manyara Ranch (MR). BWMA and RWMA are managed by member villages and receive income through tourism activities (Wilfred 2010; USAID 2013). Manyara Ranch (MR) is a multiple-use area that combines wildlife conservation with pastoralism by allowing dry season grazing access for adjacent communities (especially pastoralists from the villages Esilalei and Oltukai) (Kiffner et al. 2020). People residing in this central part of our study area (i.e. Monduli and Babati districts) mostly belong to the Kisongo Maasai or WaArusha ethnicities (Appendix S2). Both ethnicities are *Maa* speakers and part of the regional Maasai economy and society, but they also have some enduring sociocultural differences. While farming is increasingly being practiced in this semi-arid landscape (Msoffe et al. 2011), pastoralism is widespread and is the primary livelihood strategy. This is also reflected in the relatively large livestock herd sizes in Babati and Monduli compared to Karatu district (Appendix S2). The Tarangire Ecosystem still supports long-distance migrations of multiple wildlife species (e.g. wildebeest and zebra populations spend c. half of the year outside protected areas) and multiple wildlife species occupy village lands throughout the year (Gereta et al. 2004; Msoffe et al. 2010; Morrison & Bolger 2012; Kiffner et al. 2016).

In the southern part of the Tarangire Ecosystem (Kiteto District), we surveyed residents of one village located in Makame Wildlife Management Area (MWMA). The WMA generates income through trophy hunting and carbon credits. There, Maasai largely maintain traditional seasonal grazing practices (Kriegel et al. 2021) and keep large livestock herds (Appendix S2). Makame hosts a species-rich large mammal assemblage (Baker et al. 2022).

## Survey and respondent-related variables

We conducted the questionnaire surveys in 2017 (Babati, Karatu, and northern parts of Monduli district), 2019 (Kiteto district) and 2020 (southern parts of Monduli district). In 2020, we ensured that Covid-prevention protocols were followed in accordance with government guidelines (i.e. implementing social distancing, and conducting interviews exclusively outside).

To assess if participants could correctly identify each of the target species, we presented a colour photo plate depicting each species and asked interviewees to name the species in either *kiSwahili* or *Maa*. Based on the response, we assessed if the interviewee could correctly or partially (e.g. “antelope” instead of the more specific answer “impala”) identify the species or could not provide a correct name (Raycraft et al. 2025). To gauge the type of perceived costs associated with each species, we categorized answers as “none”, “tangible” (i.e. crop damages; livestock depredation, property damages), and “intangible” (i.e. threat to human health, pathogen transmission, damages to the environment). Similarly, we categorized answers to the benefit question as “none”, “tangible” (i.e. meat/skin/body part supply), and “intangible” (i.e. cultural value, aesthetic value; important ecological role etc.). We decided to classify “tourism” as an additional category because it was not always clear if the interviewee benefited directly from wildlife-based tourism. When interviews mentioned both tangible and intangible costs and benefits, we classified responses as “multiple” costs or benefits.

## Human foot print index (HFI)

As a proxy for human land use, we used the HFI dataset by Kennedy et al. (2019). This index provides a cumulative, continuous, and recent (median year 2016) measure of human landscape modification based on 13 anthropogenic stressors in five categories:

1. human settlement: population density and built-up areas
2. agriculture: cropland and livestock
3. transportation: major roads, minor roads, two-tracks, and railroads
4. mining and energy production: mining / industrial areas, oil wells and wind turbines
5. electrical infrastructure: powerlines and night-time lights

The HFI ranges from 0 to 1, with 0 representing no human modification and 1 a completely modified area.

**Appendix S2.** Summary statistics of data associated with respondents sampled in northern Tanzania, stratified by district.

|  | **Babati** | **Karatu** | **Kiteto** | **Monduli** | **Total** |
| --- | --- | --- | --- | --- | --- |
| **Village names (number of interviews)** | Kakoi (32), Ngolee (38), Olasiti (36), Vilima Vitatu (38) | Bashay (21), Chem Chem (26), Kainam (27), Rhotia Juu (29), Tloma (24) | Makame (46) | Baraka (17), Esilalei (17), Jangwani (29), Lemooti (25), Lengoolwa (25), Lolkisale (25), Losirwa (28), Makuyuni (24), Mswakini Chini (28), Mswakini Juu (22), Mugere (23), Nafco (25), Naitolia (25), Oldonyo (25), Oltukai (25) | 25 villages (680) |
| **Ethnicity (n, %)** |  |  |  |  |  |
| Iraqw | 16 (11%) | 112 (88%) | 0 (0%) | 21 (6%) | 149 (22%) |
| Maasai | 33 (23%) | 0 (0%) | 46 (100%) | 209 (58%) | 288 (42%) |
| Other | 75 (52%) | 15 (12%) | 0 (0%) | 44 (12%) | 134 (20%) |
| WaArusha | 20 (14%) | 0 (0%) | 0 (0%) | 89 (25%) | 109 (16%) |
| **Demographics** |  |  |  |  |  |
| Mean age in years (range) | 39 (18-85) | 41 (18-88) | 46 (18-90) | 41 (18-80) | 41 (18-90) |
| Gender ratio (F:M) | 1.88 (94:50) | 0.90 (60:67) | 0.39 (13:33) | 0.62 (140:223) | 0.82 (307:373) |
| **Education (n; %)** |  |  |  |  |  |
| None | 25 (17%) | 12 (9%) | 24 (53%) | 163 (45%) | 224 (33%) |
| Primary | 99 (69%) | 79 (62%) | 19 (42%) | 153 (42%) | 350 (52%) |
| Secondary and above | 20 (14%) | 36 (28%) | 2 (4%) | 47 (13%) | 105 (15%) |
| **Wealth** |  |  |  |  |  |
| Mean acres of crops (range) | 7.1 (0-100) | 2.6 (0-12) | 18.1 (0-200) | 9.3 (0-100) | 8.2 (0-200) |
| Number of assets (mean; range) | 1.8 (0-5) | 1.9 (0-5) | 0.8 (0-3) | 1.3 (0-5) | 1.5 (0-5) |
| TLU  (mean; range) | 9.1 (0-110) | 2 (0-25) | 69.6 (0-665) | 20.9 (0-170.6) | 18.2 (0-665) |
| **Spatial variables** |  |  |  |  |  |
| Mena distance (km) to PA (range) | 1.8 (0.2-4.0) | 4.1 (0.3-8.0) | 0 (0-0) | 7.4 (0-28.3) | 5.1 (0-28.3) |
| Mean HFI (range) | 0.45 (0.32-0.62) | 0.77 (0.66-0.77) | 0.60 (0.60-0.62) | 0.62 (0.37-0.72) | 0.61 (0.32-0.85) |

**Appendix S3.** Questionnaire used to survey residents of northern Tanzania about preferred wildlife management options.

| Interviewer name | |  | | Village | |  | Easting | |  | Southing | |  | | Age | |  | |
| --- | --- | --- | --- | --- | --- | --- | --- | --- | --- | --- | --- | --- | --- | --- | --- | --- | --- |
| Gender | |  | | Religion | |  | Highest Education | |  | Ethnicity | |  | | # in household | |  | |
| # Cattle | |  | | # Shoats | |  | # Donkeys | |  | # Dogs | |  | | Acres agriculture | |  | |
| # Bicycle | |  | | # Motorcyle or vehicle | |  | # Sofa set | |  | # Radio | |  | | # TV | |  | |
| Species | Can you name this species?^[[1]](#footnote-1)^ | | How often do you see this species? ^[[2]](#footnote-2)^ | | Rate the aesthetics of the species: Neutral, beautiful, ugly? | | | Are you afraid of this species? If yes, why? | Is this species causing damage? If yes, what kind?^[[3]](#footnote-3)^ | | Do you obtain benefits from this species? What kind of?^[[4]](#footnote-4)^ | | What management do you suggest? ^[[5]](#footnote-5)^ | | Who should implement management?^[[6]](#footnote-6)^ | |  |
| Elephant |  | |  | |  | | |  |  | |  | |  | |  | |  |
| Giraffe |  | |  | |  | | |  |  | |  | |  | |  | |  |
| Buffalo |  | |  | |  | | |  |  | |  | |  | |  | |  |
| Zebra |  | |  | |  | | |  |  | |  | |  | |  | |  |
| Wildebeest |  | |  | |  | | |  |  | |  | |  | |  | |  |
| Impala |  | |  | |  | | |  |  | |  | |  | |  | |  |
| Lion |  | |  | |  | | |  |  | |  | |  | |  | |  |
| Leopard |  | |  | |  | | |  |  | |  | |  | |  | |  |
| Hyena |  | |  | |  | | |  |  | |  | |  | |  | |  |
| Cheetah |  | |  | |  | | |  |  | |  | |  | |  | |  |
| Jackal |  | |  | |  | | |  |  | |  | |  | |  | |  |
| Honey badger |  | |  | |  | | |  |  | |  | |  | |  | |  |

**Appendix S4.** Summary of the Bayesian categorical model testing the effects of perceived costs and benefits and spatial variables (HFI [human footprint index], PA distance [distance to nearest protected area]) on preferred wildlife management options among rural residents of northern Tanzania. The model estimates the probability of selecting one of four management options: “None”, “Compensate”, “Prevent” or “Reduce”. The model was fitted using a multilevel Bayesian approach with random intercepts for respondents, species and villages. Estimates represent posterior means with standard errors (Est. Error) and 95% credible intervals (L-95% CrI; U-95% CrI). Rhat values close to 1 suggest good convergence of sampling. Bulk_ESS and Tail_ESS measure effective sample size in the bulk and tails of the posterior distributions, with higher values indicating better estimation stability. Random effects for respondents (SD_Compensate_ = 2.75, SD_Prevent_ = 1.81, SD_Reduce_ = 2.05) nested within villages (SD_Compensate_ = 3.36, SD_Prevent_ = 2.35, SD_Reduce_ = 2.57) and species (SD_Compensate_ = 1.62, SD_Prevent_ = 0.58, SD_Reduce_ = 1.44) indicate substantial variation.

|  | Estimate | Est. Error | l-95% CrI | u-95% CrI | Rhat | Bulk_ESS | Tail_ESS |
| --- | --- | --- | --- | --- | --- | --- | --- |
| Compensate: PA Distance (medium vs. small) | -4.74 | 1.46 | -7.65 | -1.93 | 1.00 | 3505 | 6545 |
| Compensate: PA Distance (large vs. small) | -5.39 | 1.64 | -8.68 | -2.29 | 1.00 | 3762 | 6858 |
| Compensate: HFI (medium vs. small) | 0.87 | 0.79 | -0.67 | 2.43 | 1.00 | 6234 | 9221 |
| Compensate: HFI (large vs. small) | -0.50 | 1.04 | -2.52 | 1.56 | 1.00 | 4758 | 7912 |
| Compensate: Cost (multiple vs. intangible) | 2.27 | 0.40 | 1.50 | 3.05 | 1.00 | 12224 | 12116 |
| Compensate: Costs (none vs. intangible) | -4.03 | 0.37 | -4.75 | -3.31 | 1.00 | 11964 | 12486 |
| Compensate: Cost (tangible vs. intangible) | 2.15 | 0.31 | 1.55 | 2.76 | 1.00 | 11101 | 12103 |
| Compensate: Benefit (multiple vs. mntangible) | 0.81 | 1.23 | -1.58 | 3.27 | 1.00 | 6679 | 10216 |
| Compensate: Benefit (none vs. intangible) | 0.11 | 0.95 | -1.70 | 2.03 | 1.00 | 5157 | 8581 |
| Compensate: Benefit (tangible vs intangible) | -0.19 | 1.20 | -2.54 | 2.18 | 1.00 | 6660 | 10256 |
| Compensate: Benefit (tourism vs. intangible) | 0.09 | 0.97 | -1.75 | 2.05 | 1.00 | 5104 | 8314 |
| Prevent: PA Distance (medium vs. small) | -0.12 | 0.80 | -1.69 | 1.43 | 1.00 | 2963 | 5485 |
| Prevent: PA Distance (large vs. small) | 0.26 | 0.87 | -1.47 | 1.95 | 1.00 | 3271 | 5285 |
| Prevent: HFI (medium vs. small) | -0.05 | 0.41 | -0.86 | 0.77 | 1.00 | 5683 | 8439 |
| Prevent: HFI (large vs. small) | -0.23 | 0.60 | -1.41 | 0.94 | 1.00 | 5055 | 8006 |
| Prevent: Cost (multiple vs. intangible) | 1.08 | 0.26 | 0.57 | 1.60 | 1.00 | 12121 | 13012 |
| Prevent: Cost (none vs. intangible) | -4.23 | 0.23 | -4.69 | -3.77 | 1.00 | 10912 | 12281 |
| Prevent: Cost (tangible vs. intangible) | 1.60 | 0.21 | 1.19 | 2.00 | 1.00 | 10897 | 12097 |
| Prevent: Benefit (multiple vs. intangible) | 1.71 | 0.63 | 0.49 | 2.94 | 1.00 | 6166 | 10005 |
| Prevent: Benefit (none vs. intangible) | 0.72 | 0.48 | -0.24 | 1.65 | 1.00 | 4852 | 8165 |
| Prevent: Benefit (tangible vs. intangible) | 0.85 | 0.56 | -0.25 | 1.94 | 1.00 | 5387 | 9981 |
| Prevent: Benefit (tourism vs, intangible) | 0.95 | 0.49 | -0.02 | 1.91 | 1.00 | 4851 | 8056 |
| Reduce: PA Distance (medium vs. small) | -3.76 | 1.30 | -6.38 | -1.27 | 1.00 | 3279 | 5886 |
| Reduce: PA Distance (large vs. small) | -3.36 | 1.38 | -6.13 | -0.71 | 1.00 | 3311 | 6087 |
| reduce: HFI (medium vs. small) | 0.30 | 0.61 | -0.89 | 1.50 | 1.00 | 6823 | 10316 |
| reduce: HFI (large vs. small) | -1.12 | 0.78 | -2.68 | 0.41 | 1.00 | 6084 | 9642 |
| Reduce: Cost (multiple vs. intangible) | 1.57 | 0.36 | 0.86 | 2.27 | 1.00 | 11637 | 12287 |
| Reduce: Cost (none vs. intangible) | -4.44 | 0.42 | -5.27 | -3.63 | 1.00 | 12942 | 12375 |
| Reduce: Cost (tangible vs. intangible) | 1.64 | 0.30 | 1.04 | 2.23 | 1.00 | 11623 | 12273 |
| Reduce: Benefit (multiple vs. intangible) | 2.29 | 1.19 | -0.01 | 4.67 | 1.00 | 5808 | 8912 |
| Reduce: Benefit (none vs. intangible) | 1.51 | 0.92 | -0.19 | 3.38 | 1.00 | 4405 | 6991 |
| Reduce: Benefit (tangible vs. intangible) | 1.88 | 1.02 | -0.06 | 3.93 | 1.00 | 4623 | 7243 |
| Reduce: Benefit (tourism vs. intangible) | -0.57 | 0.96 | -2.35 | 1.38 | 1.00 | 4413 | 7315 |

**Appendix S5**. Proportions of respondents expressing fear toward six herbivore (grey bars) and six carnivore (black bars) species, based on questionnaires conducted in rural parts of northern Tanzania. Numbers next to the bars indicate the count of the fear responses (numerator) out of the total number of valid responses (denominator; i.e. number of respondents who were able to at least partially identify the species).


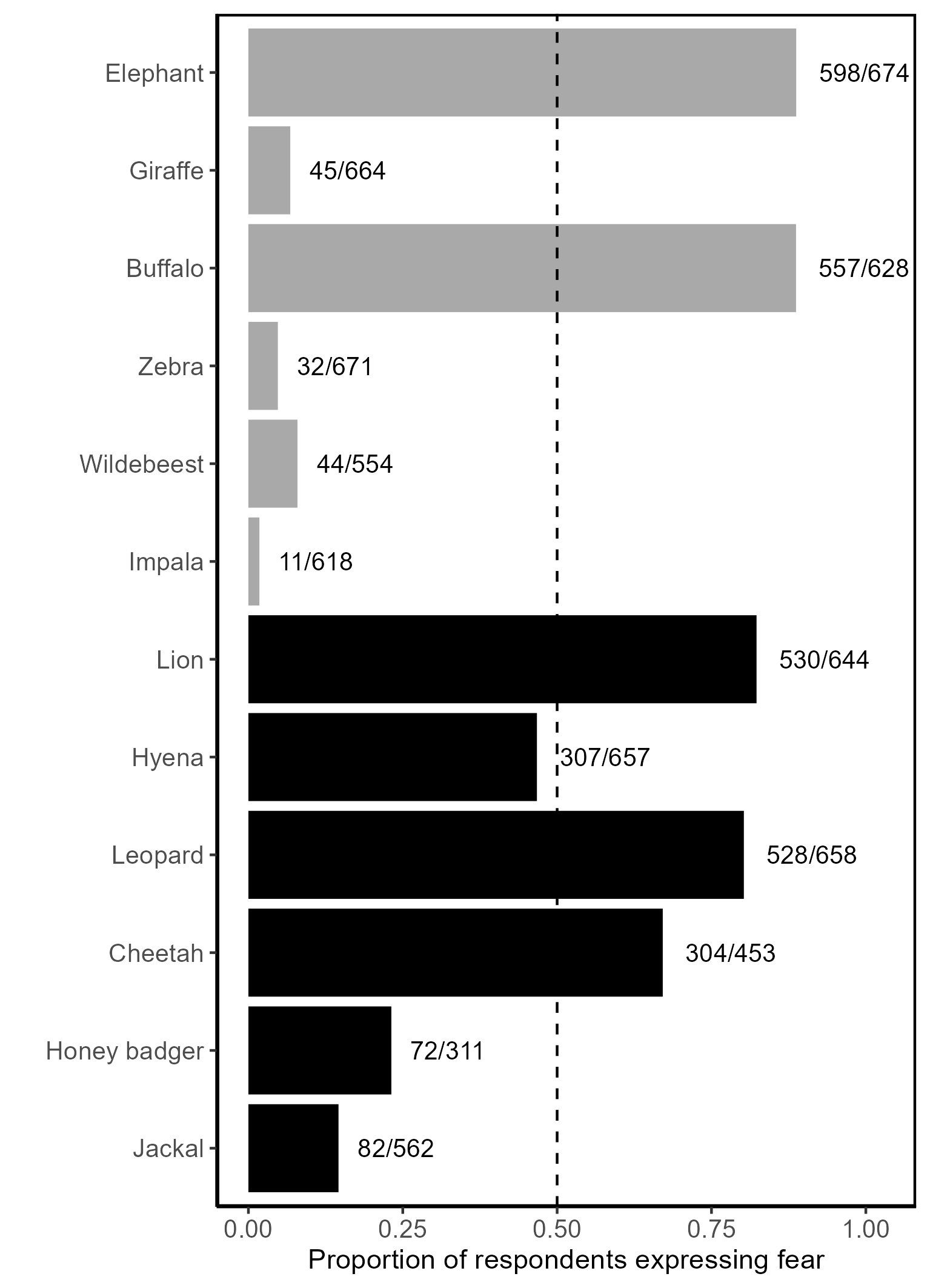


**References**

Baker M, Anderson SJ, Kiffner C. 2022. Financing conservation in the 21st century - Investing in nature-based climate solutions in Makame Wildlife Management Area. Pages 319-337 in C. Kiffner, M. L. Bond, and D. E. Lee (editors). Tarangire: Human-Wildlife Coexistence in a Fragmented Ecosystem. Springer International Publishing, Cham.

Diplock N, Johnston K, Mellon A, Mitchell L, Moore M, Schneider D, Taylor A, Whitney J, Zegar K, Kioko J, Kiffner C. 2018. Large mammal declines and the incipient loss of mammal-bird mutualisms in an African savanna ecosystem. PLOS ONE **13**:e0202536.

Gereta E, Meing’ataki GEO, Mduma S, Wolanski E. 2004. The role of wetlands in wildlife migration in the Tarangire ecosystem, Tanzania. Wetlands Ecology Management **12**:285–299.

Homewood KM, Rodgers WA. 2004. Maasailand ecology: Pastoralist development and wildlife conservation in Ngorongoro, Tanzania. Cambridge University Press, Cambridge.

Kennedy CM, Oakleaf JR, Theobald DM, Baruch-Mordo S, Kiesecker J. 2019. Managing the middle: A shift in conservation priorities based on the global human modification gradient. Global Change Biology **25**:811–826.

Kiffner C, Kioko J, Baylis J, Beckwith C, Brunner C, Burns C, Chavez-Molina V, Cotton S, Glazik L, Loftis E, Moran M, O'Neill C, Theisinger O, Kissui B,Kioko J. 2020. Long-term persistence of wildlife populations in a pastoral area. Ecology and Evolution **10**:10000–10016.

Kiffner C, Nagar S, Kollmar C, Kioko J. 2016. Wildlife species richness and densities in wildlife corridors of Northern Tanzania. Journal for Nature Conservation **31**:29–37.

Kriegel ER, Cherney DJR, Kiffner C. 2021. Conventional knowledge, general attitudes and risk perceptions towards zoonotic diseases among Maasai in northern Tanzania. Heliyon **7**:e07041.

Morrison TA, Bolger DT. 2012. Wet season range fidelity in a tropical migratory ungulate. Journal of Animal Ecology **81**:543–552.

Msoffe FU, Kifugo SC, Said MY, Neselle MO, Van Gardingen P, Reid RS, Ogutu JO, Herero M, de Leeuw J. 2011. Drivers and impacts of land-use change in the Maasai Steppe of northern Tanzania: an ecological, social and political analysis. Journal of Land Use Science **6**:261–281.

Msoffe FU, Ogutu JO, Kaaya J, Bedelian C, Said MY, Kifugo SC, Reid RS, Neselle M, Van Gardingen P, Thirgood S. 2010. Participatory wildlife surveys in communal lands: A case study from Simanjiro, Tanzania. African Journal of Ecology **48**:727–735.

Prins HHT, Loth PE. 1988. Rainfall patterns as background to plant phenology in northern Tanzania. Journal of Biogeography **15**: 451–463.

Raycraft J, Becchina R, Bettermann D, Koester S, Kriegel E, Lindsay K, Ole EM, Ramirez E, Spizuco B, Kiffner C. 2025. Socio-ecological correlates of wildlife species identification across rural communities in northern Tanzania. People and Nature **7**:2002-2018.

USAID. 2013. Tanzania wildlife management areas (WMA) evaluation - final evaluation report.

Wilfred P. 2010. Towards sustainable Wildlife Management Areas in Tanzania. Tropical Conservation Science **3**:103–116.

1. 0=no / false; 1=almost correct; 2=correct [↑](#footnote-ref-1)
2. Daily=365; Weekly=56; Monthly=12; Once a year=1; Never=0 [↑](#footnote-ref-2)
3. Damage to crops, livestock, pet, property, disease, injury [↑](#footnote-ref-3)
4. Tourism, meat, other (specify) [↑](#footnote-ref-4)
5. Nothing=0; 1=Preventing damage e.g. by fencing; 3=compensation of damages; 4=reduce population size [↑](#footnote-ref-5)
6. Ourselves=1; 2=local authorities; 3=government; 4=NGOs; 5=others (specify) [↑](#footnote-ref-6)
